# Enhanced Sunlight-Powered Photocatalysis and Methanol Oxidation Activities of Co_3_O_4_-Embedded Polymeric Carbon Nitride Nanostructures

**DOI:** 10.3390/nano13182508

**Published:** 2023-09-06

**Authors:** Surya Veerendra Prabhakar Vattikuti, J. Pundareekam Goud, P. Rosaiah, P. Reddy Prasad, Ammar M. Tighezza, Jaesool Shim

**Affiliations:** 1 School of Mechanical Engineering, Yeungnam University, Gyeongsan 38541, Republic of Korea; 2 Department of Physics, Koneru Lakshmaiah Education Foundation, Bowrampet, Hyderabad 500043, Telangana, India; 3 Department of Physics, Saveetha School of Engineering, Saveetha Institute of Medical and Technical Sciences (SIMATS), Thandalam, Chennai 602105, Tamil Nadu, India; 4 Department of Chemistry, Institute of Aeronautical Engineering, Hyderabad 500043, Telangana, India; 5 Department of Chemistry, College of Science, King Saud University, Riyadh 11451, Saudi Arabia

**Keywords:** photocatalysts, solar energy, dye degradation, pollutants, 2D nanosheets, metal oxide

## Abstract

The contamination of water by organic substances poses a significant global challenge. To address these pressing environmental and energy concerns, this study emphasizes the importance of developing effective photocatalysts powered by sunlight. In this research, we achieved the successful synthesis of a novel photocatalyst comprised of polymeric carbon nitride (CN) nanosheets embedded with Co_3_O_4_ material, denoted as CN-CO. The synthesis process involved subjecting the mixture to 500 °C for 10 h in a muffle furnace. Structural and morphological analyses confirmed the formation of CN-CO nanostructures, which exhibited remarkable enhancements in photocatalytic activity for the removal of methylene blue (MB) pollutants under replicated sunlight. After 90 min of exposure, the degradation rate reached an impressive 98.9%, surpassing the degradation rates of 62.3% for pure CN and 89.32% for pure Co_3_O_4_ during the same time period. This significant improvement can be attributed to the exceptional light captivation capabilities and efficient charge separation abilities of the CN-CO nanostructures. Furthermore, the CN-CO nanostructures demonstrated impressive photocurrent density-time (j-t) activity under sunlight, with a photocurrent density of 2.51 μA/cm^2^ at 0.5 V. The CN-CO nanostructure exhibited excellent methanol oxidation reaction (MOR) activity with the highest current density of 83.71 mA/cm^2^ at an optimal 2 M methanol concentration, benefiting from the synergy effects of CN and CO in the nanostructure. Overall, this study presents a straightforward and effective method for producing CN-based photocatalysts decorated with semiconductor nanosized materials. The outcomes of this research shed light on the design of nanostructures for energy-related applications, while also providing insights into the development of efficient photocatalytic materials for addressing environmental challenges.

## 1. Introduction

The presence of organic pollutants, such as commonly used dyes and pigments, in industrial wastewater discharges poses a significant environmental pollution problem [1,2]. Many of these contaminants are non-biodegradable and have the potential to be carcinogenic. For instance, MB is extensively employed in various industries, including food, paper, wool, silk, pharmaceutical, and cosmetics, further exacerbating the issue [3]. Wastewater containing cationic thiazine dyes is a significant source of water pollution, as these dyes impede light penetration and disrupt photosynthetic reactions in aquatic plants, consequently impacting the aquatic ecosystem. Moreover, these synthetic dyes are classified as carcinogenic and toxic chemicals, posing a serious risk to both humans and aquatic organisms [4]. To address these environmental concerns, heterogeneous photocatalysis has emerged as a promising technology. It offers the potential to degrade pollutants into harmless inorganic substances such as CO_2_ and H_2_O, while also enabling useful chemical conversions of byproducts. Photocatalysis has gained considerable attention due to its high efficiency, low cost, and the utilization of abundant solar energy as the primary energy source [5].

Transition metal oxides efficiently act as photocatalysts even under optimal environments with no added energy input required [6] in energy evolution/storage applications [7,8,9,10]. Among these, cobalt-type photocatalysts have shown enhanced photodegradation of organic dyes and antibodies compared to other transition metal oxides [11], thanks to their narrow band gap positions and chemical stability. Co_3_O_4_ has been broadly employed as a photocatalyst for the removal of organic pollutants [12]. However, its dye degradation effectiveness is inadequate because of factors such as deprived dispersion and rapid recombination rates. Moreover, considering that cobalt is an affluent and erratic metal, several investigation studies and developments have focused on reducing and immobilizing the cobalt concentration to diminish leaching [13].

Polymeric carbon nitride (CN) materials have gained similar research interest for their beneficial properties including stability, nontoxicity, strong visible-light absorption with a narrow bandgap of 2.7 eV, and favorable electrical characteristics [14]. The ease of synthesis CN subsequently favors their applications. CN can be certainly generated by updraft pyrolysis/condensation of common N-rich reagents including melamine and urea [15]. CN-based materials are widely investigated for their prospective photocatalytic applications including, photodegradation of pollutant/organic dye removal or decomposition of organic contaminants and H_2_O splitting as well as CO_2_ reduction [16]. CN can also transfer photoinduced e^−^ to another material by the CB because of the interior electric potential alteration [17]. Indeed, the photocatalytic efficiency of CN materials is limited owing to the fast recombination of charge carriers. To overcome this issue, researchers have extensively explored enhancing CN’s performance by introducing coupling with other metal oxides [18,19,20,21,22]. Recently, CN/SnO_2_ [18], CN/Co_3_O_4_ [19,20], CN/NiO [21], and CN/MnCo_2_O_4_ [22] composites have been reported with improved photoactivity ascribed to the role of the CN in better separation of the charge carriers responsible for catalytic activity [23]. Commonly, the design of highly efficient heterojunction catalysts focuses on achieving ideal interfaces between semiconductors to enhance charge transfer and separation [24,25]. In the case of Co_3_O_4_ and g-C_3_N_4_ nanostructures, their composite structures exhibit a large surface area, facilitating intimate interfaces that promote rapid and effective charge transfer and separation. Therefore, the combination of Co_3_O_4_ and g-C_3_N_4_ in these nanostructures provides favorable conditions for efficient catalytic activity.

In this work, we present the production of Co_3_O_4_ nanoparticles embedded in CN nanosheets (referred to as CN-CO) through a direct precursor pyrolysis process. This method presents a unique combination of materials, specifically CN nanosheets embedded with Co_3_O_4_. The synthesized CN-CO nanostructure was extensively characterized using techniques such as XRD, FESEM, HRTEM, BET, DRS, XPS, and PL. We investigated the photocatalytic applications of CN-CO, including the degradation of methylene blue (MB), and evaluated its performance in the MOR. The CN-CO nanostructure exhibited excellent chemical stability and durability, as demonstrated by multiple recycling trials in photocatalysis. This hybrid photocatalyst offers distinct properties compared to existing materials and holds promise for enhancing photocatalytic performance. Furthermore, we proposed a photocatalytic mechanism for the degradation of MB by CN-CO. Additionally, we evaluated the MOR activity of CN-CO, and the results revealed that the highest current density of 83.71 mA/cm^2^ was achieved with the addition of 2 M methanol in a 1 M KOH. Overall, this work provides insights into the synthesis, characterization, and photocatalytic applications of CN-CO nanostructures, highlighting their potential in both environmental remediation and energy conversion processes.

## 2. Materials Section

### 2.1. Materials

Melamine and cobalt nitrate hexahydrate were procured from DAEJUNG, Republic of Korea, and utilized without undergoing additional treatment processes.

### 2.2. Synthesis of CN-CO Nanostructure

**Figure 1:**
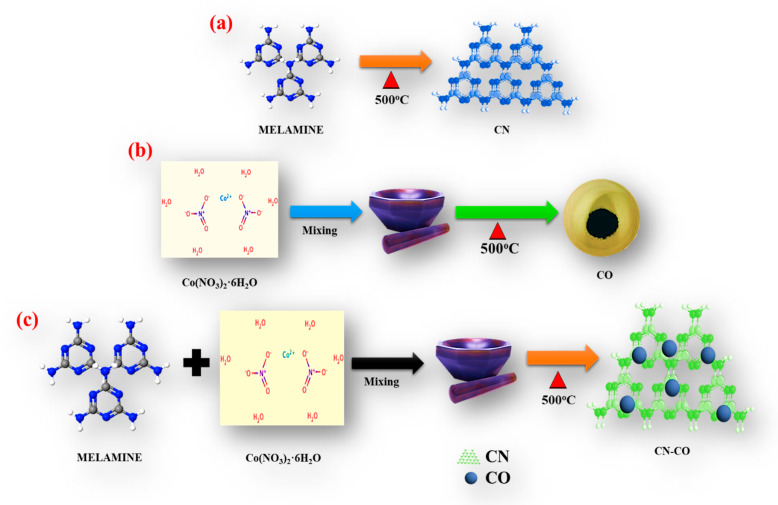
Representation of synthetic procedure of (**a**) CN, (**b**) CO, and (**c**) CN-CO nanostructure.

Figure 1a–c shows the reprehensive schematic of the CN, CO, and CN-CO nanostructure preparation process. The optimized CN-CO nanostructures reported here were produced by grinding a mixture of melamine and cobalt precursors with a weight-to-weight ratio of 1:0.20 (Figure 1c). Synthesis of CN and CO followed the same protocol (Figure 1a,b). The finished products were cleaned and dried at 100 °C for an over-night. Please see the Supplementary Materials for more details on material characterization and photocatalytic/photo-electrochemical testing.

## 3. Results and Discussion

### 3.1. Description of CN, CO and CN-CO Nanostructure

#### 3.1.1. Phase Structure

**Figure 2:**
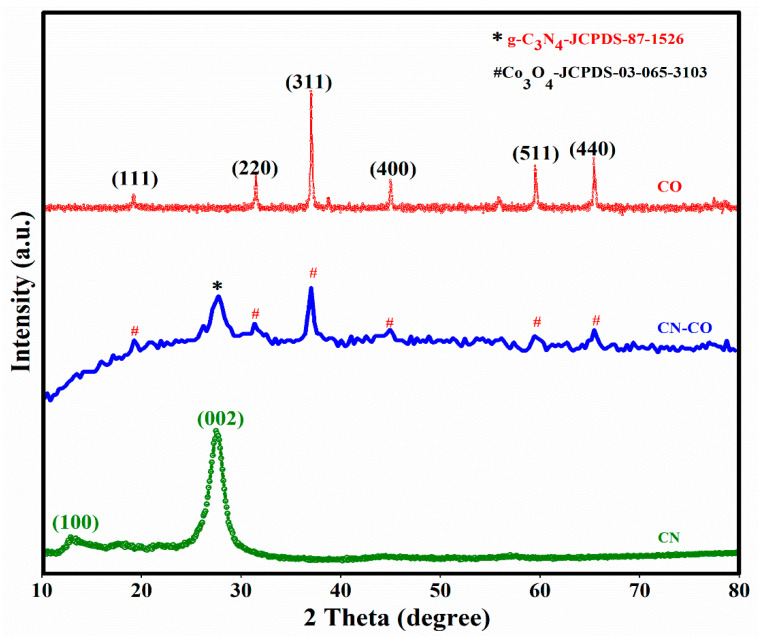
XRD pattern of CN, Co, and CN-CO nanostructure.

The XRD profiles of the CN, CO, and CN-CO nanostructures (Figure 2). The CN sample exhibits a characteristic peak at 27.38° (002), indicating the interlayer stacking of aromatic groups, and a peak at 13.1° (100) resulting in the in-plane operational packing of carbon nitride [26]. The CO sample shows diffraction peaks attributed to the Co_3_O_4_ phase (JCPDS-03-065-3103). The peaks at 2θ values of 19.15°, 31.53°, 37.07°, 44.9°, 59.54°, and 65.37° can be allotted to the (111), (220), (311), (400), (511), and (440) planes of the cubic-type structure of Co_3_O_4_, respectively. In the XRD pattern of the CN-CO nanostructure, both CN and CO peaks are observed. However, the intensity of the CN peaks decreases upon incorporation of CO, indicating the coexistence of CN and CO in the CN-CO nanostructure. The absence of additional impurity peaks suggests that the synthesized CN-Co nanostructures are pure and stable.

#### 3.1.2. Morphological Structure and Composition

**Figure 3:**
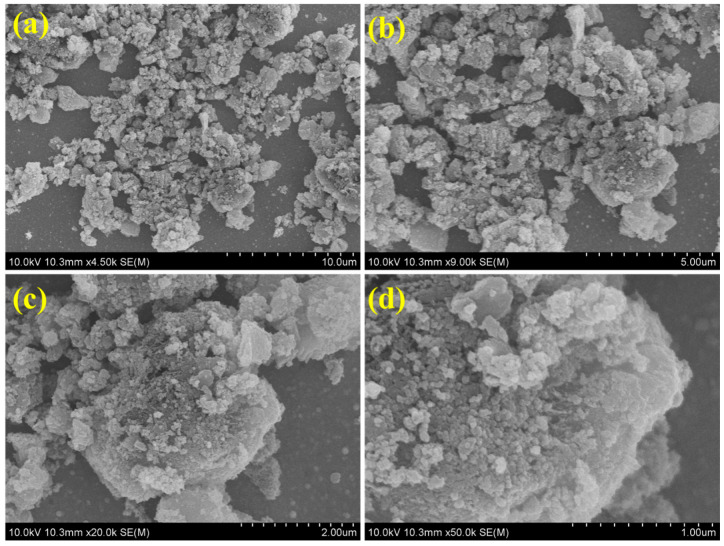
(**a**–**d**) FESEM images of the CN-CO nanostructure.

**Figure 4:**
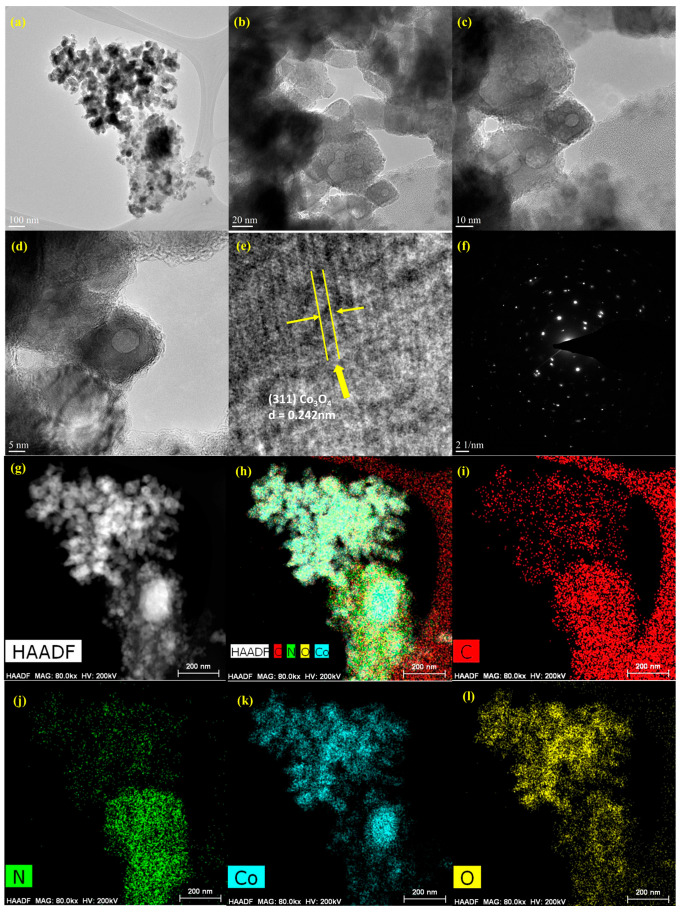
(**a**–**e**) HRTEM images, (**f**) SAED pattern, (**g**) HAADF image, (**h**) HAADF with combined mapping elements, and (**i**–**l**) individual elemental mapping of C, N, Co, and O traces in the CN-CO nanostructure.

The exterior morphologies of the CN-CO nanostructure were inspected using FESEM (Figure 3). The CN materials exhibit a layered structure with multiple stacking layers, consistent with previous reports [27]. The FESEM images of the CN-CO nanostructure illustrate the agglomeration of CO on the surface of CN, indicating the successful formation of a composite structure. Furthermore, HRTEM analysis was accomplished on the CN-CO nanostructure (Figure 4a–d) and corresponding lattice fringes (Figure 4e). The images clearly depict small CO nanoparticles dispersed randomly on the CN layers. The close and intimate interfaces between CN and CO are anticipated to play a crucial role in enhancing the photocatalytic activity by facilitating the formation of an interior electric arena at their interfaces. Additionally, the SAED pattern of the CN-CO nanostructure (Figure 4f) exhibits a distinct modulated diffraction pattern, indicating the preferential alignment of the nanoparticles. This alignment is likely a result of the interactions between neighboring nanoparticles, including van der Waals forces or dipolar interactions, as also confirmed by the HAADF image (Figure 4g). The SAED results align well with the XRD analyses, confirming the attendance of C, N, Co, and O traces (Figure 4h–l). Figure 4h displays the combined elemental mapping images of the CN-CO nanostructure, revealing the coexistence of CN and CO materials. Individual elemental mapping images (Figure 4i–l). Finally, the elemental mapping analysis provides further evidence for the presence of CO on the surface of CN.

#### 3.1.3. XPS Studies

**Figure 5:**
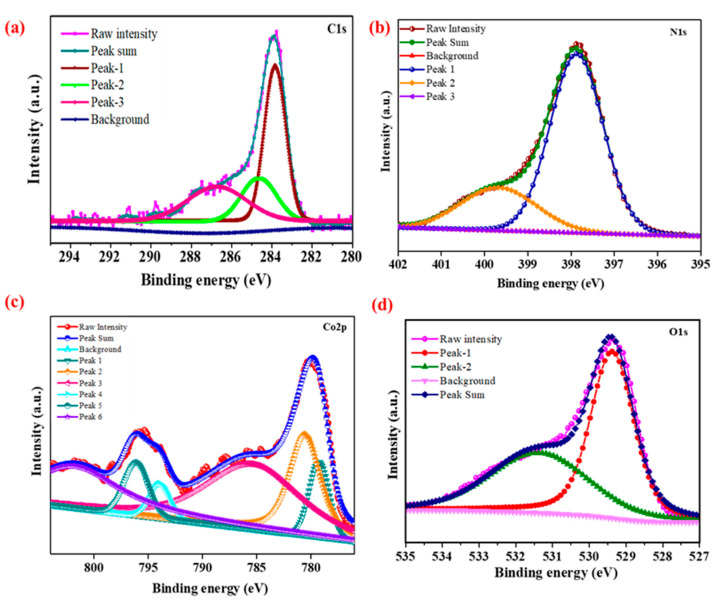
XPS spectra of (**a**) C1s, (**b**) N1s, (**c**) Co2p, and (**d**) O1s of the CN-CO nanostructure.

Figure 5a presents the C1s XPS pattern of the CN-CO nanostructure. The peaks observed at 284.54 eV and 288.15 eV correspond to carbon crusts and N=C-N of carbon sp2 hybridization in CN, respectively. In Figure 5b, the N1s XPS spectra exhibit major bands at 399.23 eV and 401.23 eV, consigned to the sp2 hybridized N(C-N=C) and tertiary nitrogen N-(C)_3_, individually [28,29]. Figure 5c displays the Co2p emission spectra, revealing two significant peaks conforming to Co 2p_1/2_ and Co 2p_3/2_ spin-path coupling. Satellite peaks perceived at 779.5 eV and 795.43 eV are attributed to Co^2+^, while the peaks at 782.13 eV and 797.8 eV for Co 2p_3/2_ and Co 2p_1/2_, correspondingly, are assigned to Co^3+^ [30,31,32,33]. In Figure 5d, the O1s spectra show that the oxygen lattice in the spinel structure is located at 529.84 eV, while the O_2_ peak corresponds to water at 532.8 eV [32,33,34].

#### 3.1.4. DRS Spectra

**Figure 6:**
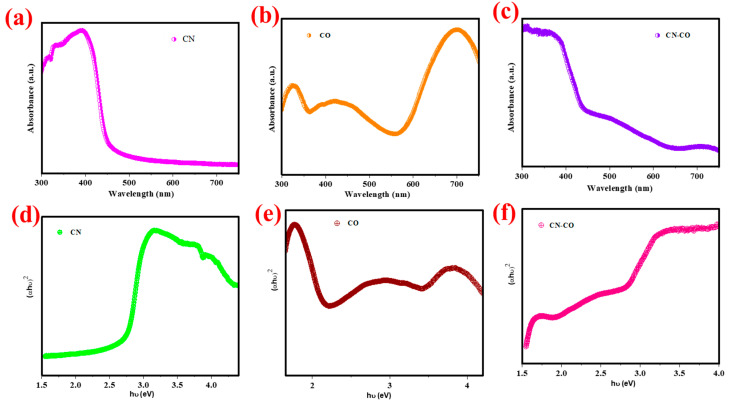
(**a**–**c**) UV-vis spectra and (**d**–**f**) Tauc’s plots of the CN, CO, and CN-CO nanostructures.

Figure 6a–c depicts the absorbance spectra of CN, CO, and the CN-CO nanostructure. All samples exhibit absorption curves in the visible range, with absorption edges ranging from 450 to 535 nm. Interestingly, the addition of CO to CN consequences in an absorption edge redshift compared to CN. This redshift is advantageous for achieving enhanced photocatalytic performance. The associated Tauc plots [35] of CN, CO, and the CN-CO nanostructure (Figure 6d–f). The calculated band gaps of CN, CO, and the CN-CO nanostructures are determined to be 2.68 eV, 2.32 eV, and 2.37 eV, respectively. Notably, the heterostructure catalyst exhibits a reduced bandgap compared to CO, which can be endorsed to the interface between CN and CO. As a result, the nanostructured photocatalyst facilitates the generation of additional photoinduced carriers under solar light illumination compared to the pure samples, leading to a substantial enhancement of photoactivity.

#### 3.1.5. Photocatalytic Activity

**Figure 7:**
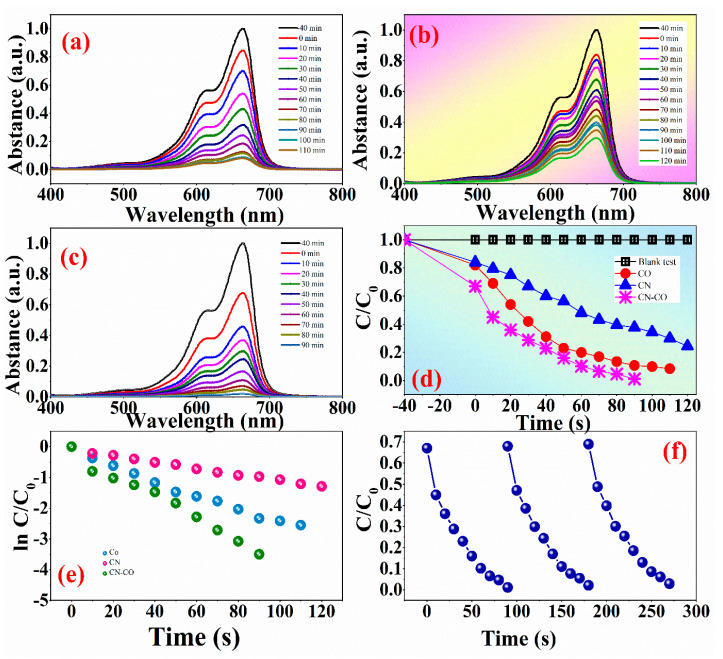
Time-dependent UV-visible spectra of MB dye in aqueous dispersion over (**a**) CO, (**b**) CN, (**c**) CN-CO nanostructure, (**d**) variation photo-degradation of MB dye over all photocatalysts, (**e**) pseudo-first-order kinetics of degradation of MB dye over all photocatalysts, and (**f**) Cycling stability of CN-CO nanostructure under identical testing conditions.

The photocatalytic degradation capabilities of CN, CO, and CN-CO nanostructures were evaluated for the removal of the MB dye. The dye removal rate was monitored over time, and the absorption graphs are presented in Figure 7a–c. During the photodegradation process, the peak intensity of the absorption spectrum corresponding to the MB dye at 668 nm progressively decreased, indicating its degradation. Prior to the testing, the MB dye and photocatalysts were thoroughly mixed and allowed to equilibrate in the obscure for 30 min to confirm proper adsorption and surface coverage. Among the photocatalysts tested, the CN-CO nanostructure exhibited superior photocatalytic activity compared to the bare catalysts. Figure 7d illustrates the decolorization efficiency of all catalysts for the MB dye solution under simulated solar light illumination. Furthermore, the self-decolorization behavior of the MB dye was tested without the catalyst, and it showed a negligible tendency for self-decolorization. After 90 min of light illumination, the dye adsorption rate over the CN-CO nanostructure reached 98.9%, whereas it was 89.32% for pristine CO and 62.3% for CN. To further investigate and compare the photoactivity of all catalysts, the kinetics of MB dye decolorization were examined, as depicted in Figure 7e. The rate constants for CN, CO, and CN-CO nanostructures were determined to be 0.249, 0.031, and 0.352 min^−1^, respectively. The results clearly indicate that the CN-CO nanostructure exhibited a higher rate constant compared to the individual catalysts, attributed to its enhanced absorption efficiency. Figure 7f demonstrates the cycling stability of the CN-CO nanostructures over three consecutive runs, showing that the CN-CO nanostructure maintained its degradation ability with only a 2.9% loss in its initial degradation rate.

The CN-CO nanostructure exhibited remarkable catalytic activity, which can be attributed to several unique and special features: (i) 2D/2D mesoporous structure: the CN-CO catalysts possessed a mesoporous structure with a two-dimensional configuration. This structure provided plentiful exposed active centers, allowing for efficient adsorption of substrates. The abundant active centers on the catalyst surface played a key role in its remarkable catalytic activity. The mesoporous structure also facilitated the reactant’s diffusion and products, enhancing the complete catalytic performance; (ii) incorporation of CO in the CN-CO nanostructure: the introduction of CO into the CN matrix created a synergistic effect that enhanced the catalytic activity. The presence of CO facilitated rapid ion transfer within the nanostructure, foremost to improve charge carrier separation and diminished recombination. This efficient charge transfers and minimal recombination of charge carriers contributed to the enhanced photoactivity of the CN-CO nanostructure; (iii) enhanced light absorption: the CN-CO nanostructure exhibited strong observable light absorption owing to the narrow bandgap of the CN material. This allowed for efficient utilization of solar energy as the driving force for catalytic reactions. The absorption of light by the nanostructure promoted the generation of photoinduced electrons and holes, which participated in various catalytic processes; (iv) synergy effects between CN and CO: the interaction between CN and CO in the nanostructure created a synergistic effect that further improved the catalytic performance. The combination of these materials provided unique electronic and structural properties, enabling efficient charge transfer and creating favorable reaction pathways. This synergy enhanced the overall catalytic activity of the CN-CO nanostructure; (v) chemical stability and durability: the CN-CO nanostructure demonstrated good chemical stability and durability during photocatalytic cycling. It maintained its catalytic activity over multiple reaction cycles without significant degradation or loss of performance. This stability ensured the long-term effectiveness of the catalyst for practical applications. Overall, the unique and special features of the CN-CO nanostructure, including its mesoporous structure, incorporation of CO, enhanced light absorption, synergistic effects, and chemical stability, collectively contributed to its notable catalytic activity and made it a promising candidate for various photocatalytic uses.

#### 3.1.6. Confirmation of Charge Transfer through j-t, EIS, PL Analysis and Scavenger Test

**Figure 8:**
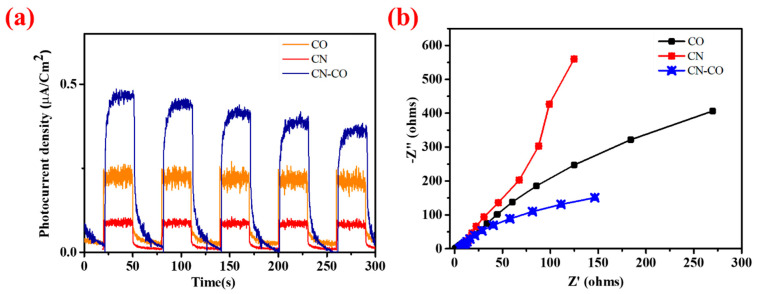
(**a**) j-t curves and (**b**) Nyquist plots of the CO, CN, and CN-CO nanostructures.

To gain further insights into the charge transport activities of the catalysts, the Photocurrent density-time (j-t) response was analyzed for CN, CO, and the CN-CO nanostructure under simulated solar light illumination in a 0.5 M Na_2_SO_4_ electrolyte, with on and off cycles. Figure 8a illustrates the j-t response of CN, CO, and the CN-CO nanostructures during the same experimental procedure. Notably, the CN-CO nanostructure photocatalyst exhibited a higher photocurrent density (0.37 μA/cm^2^) compared to the bare CN (0.065 μA/cm^2^) and CO (0.21 μA/cm^2^). This enhanced photocurrent response observed for the CN-CO nanostructure can be attributed to a lower photoinduced carrier recombination rate, indicating further capable separation and utilization of the photoinduced charge carriers. Figure 8b presents the Nyquist plot of CN, CO, and the CN-CO nanostructure. The arc radius observed on the Nyquist plot provides insights into the reaction rate arising at the electrode surface. In the plot, the arc radius of the CN-CO nanostructure is smaller than that of CO and CN, indicating a more effective separation of photoinduced carriers over the CN-CO nanostructure. This suggests that the CN-CO nanostructure facilitates efficient charge carrier separation, contributing to its enhanced photocatalytic activity.

**Figure 9:**
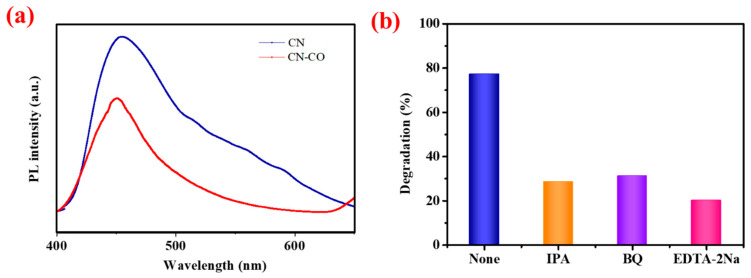
(**a**) PL spectra of the CN and CN-CO nanostructures and (**b**) effects of various scavengers on the degradation of MB under solar light irradiation.

To evaluate the separation and transferability of photoinduced carriers, PL spectra were recorded for both the CN and CN-CO nanostructures (Figure 9a). An efficient catalyst exhibits low emission intensity, indicating a lower recombination rate of the photoinduced electrons/holes responsible for the photocatalytic reaction. In the case of pure CN, an intense PL emission was observed at 455 nm, which can be attributed to the bandgap shift. However, upon the introduction of CO onto the CN surface to form the CN-CO nanostructure catalysts, the PL intensity decreased. This decrease in PL intensity suggests effective migration of photoinduced carriers within the nanostructure [36]. The presence of CO on the catalyst surface enhances the absorption of visible light, leading to improved charge carrier association within the nanostructure. As a result, the incorporation of CO into the CN matrix contributes to improved photocatalytic activity.

To elucidate the photocatalytic mechanism of the CN-CO nanostructure, we conducted trapping experiments employing EDTA-2Na, IPA, and BQ as scavengers to capture the photoinduced hole (h^+^), hydroxyl radical (•OH), and superoxide radical (•O_2_^−^), correspondingly. Photocatalytic processes are well-known to involve the catalytic participation of photoinduced e^−^ and h^+^ in the photodegradation reaction. When the incident energy surpasses the photocatalyst’s E_g_, the electrons in the VB are excited and transition to the CB, leaving behind holes in the VB. These separated e^−^ and h^+^ s subsequently migrate to the catalyst’s surface and interfaces, where they interact with O_2_ and H_2_O to harvest highly reactive oxidizing species, including h^+^, •OH, and •O_2_^−^, facilitating advanced reactions. By conducting trapping experiments using specific scavengers, the role of each reactive species can be identified. EDTA-2Na is used to scavenge photogenerated h^+^, IPA is employed to scavenge •OH, and BQ is utilized as a scavenger for •O_2_^−^. These experiments provide valuable insights into the mechanism of photocatalysis in the CN-CO nanostructure. Figure 9b illustrates the results of the trapping test using various scavengers mentioned above. The degradation rate of the CN-CO nanostructure considerably diminished with the accumulation of scavengers, particularly with IPA and EDTA-2Na. These results reveal that •OH and h^+^ play dominant species in the photocatalytic process, while •O_2_^−^ plays a secondary role. The decreased degradation rate in the presence of IPA and EDTA-2Na indicates that the scavenging of •OH and h^+^ has a pronounced impact on the overall degradation process, highlighting their importance in the photocatalytic activity of the CN-CO nanostructure.

#### 3.1.7. Photocatalytic Mechanism

**Figure 10:**
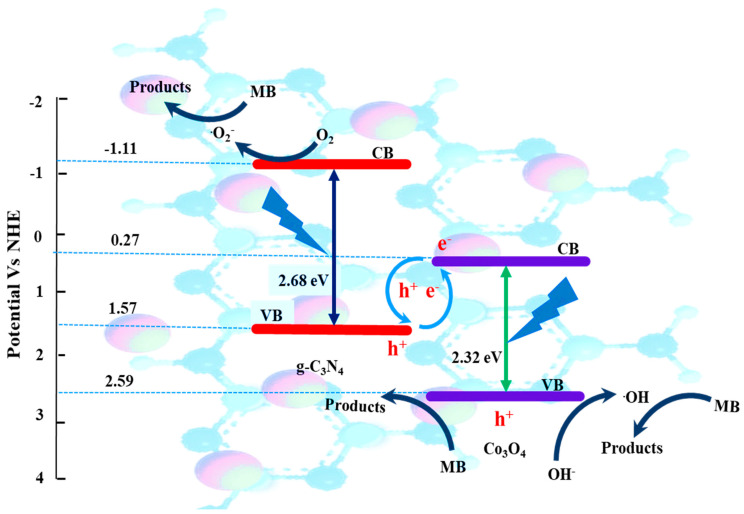
Proposed photocatalytic mechanism of MB dye degradation over the CN-CO nanostructure.

Based on the j-t, PL, and trapping results obtained, a proposed heterojunction scheme for the CN-CO nanostructure is presented in Figure 10. The CB potentials of CN and CO are determined to be −1.11 eV and 0.27 eV, correspondingly. This finding confirms that the CB electrons of CN, rather than CO, are capable of reducing dissolved O_2_ molecules in an aqueous solution to form •O_2_^−^. This is supported by the fact that the standard redox potential of O_2_/•O_2_^−^ is −0.046 eV versus the NHE [37]. In contrast, the lower VB potential of CO (2.59 eV) instead of CN (1.57 eV) enables the oxidation of OH^−^ to •OH, as indicated by the standard redox potential of •OH/OH^−^ at 2.3 eV versus NHE [38]. Active species trapping experiments have also confirmed the involvement of h^+^, •OH, and •O_2_^−^ in the degradation of MB over the CN-CO nanostructure. These experiments provide further evidence that •O_2_^−^ and •OH play imperative roles in the photocatalytic process, contributing to the efficient removal of MB contaminants by the CN-CO nanostructure. Hence, it is imperative to establish a Z-scheme system between CN and CO. When subjected to light illumination, the electrons in the VB of CN and CO undergo a transfer to the CB, resulting in the generation of holes in their respective VBs. Subsequently, a rapid recombination occurs at the interface of CO’s CB electrons and CN’s VB holes, significantly enhancing the efficiency of charge carrier separation. The accumulated electrons in the CB of CN are captured by dissolved O_2_ molecules in the aqueous solution, prominent to the production of •O_2_^−^. Additionally, the accumulated holes in the VB of CO can potentially react with OH^−^ to generate •OH. Ultimately, the activated species, namely •OH, h^+^, and •O_2_^−^, all participate in the degradation reaction of MB dye.

#### 3.1.8. Electrocatalytic Methanol Oxidation

**Figure 11:**
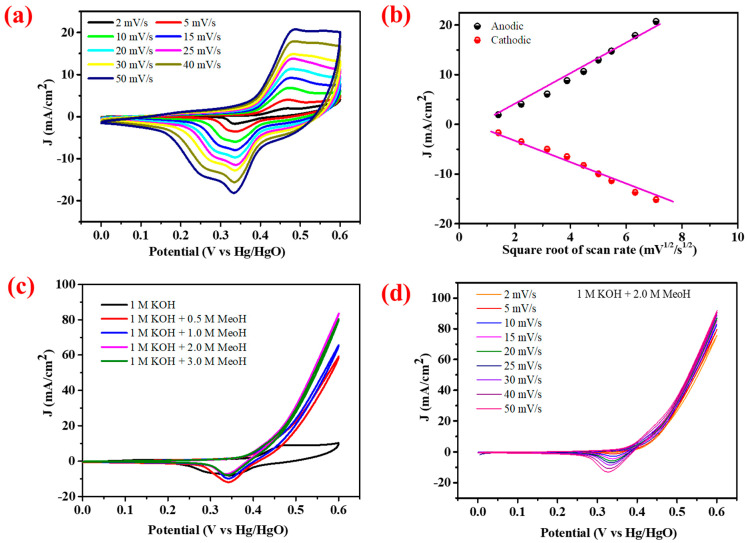
Electrochemical methanol oxidation performance: (**a**) CV curves of the CN-CO nanostructure at different scan rates, (**b**) anodic/cathodic peak current, (**c**) CV profiles without and with different methanol concentrations, and (**d**) CV profiles at various scan rates at with 2 M methanol.

The electrocatalytic performance of the CN-CO nanostructure electrode for methanol oxidation was investigated using various electrochemical techniques. The behavior of the electrode was evaluated, and Figure 11a displays the CV profiles at different sweep rates (2–50 mV/s) in a 1 M KOH before the addition of methanol. The current density value upsurges as the sweep rate upsurges, which can be attributed to the diffusion of Co^3+^ ions within the electrode. This diffusion process leads to an increase in the oxidation and reduction currents, resulting in a larger area under the curves in the CV profiles. Figure 11b illustrates the regression factors of the anodic and cathodic peak currents plotted against the sweep rates square root. The anodic/cathodic peak currents of the CN-CO nanostructure exhibit diffusional characteristics influenced by the sweep rate. The estimated electroactive surface area of the electrode is determined to be 0.43 cm^2^, indicating a substantial active surface area that facilitates rapid charge transfer at the electrode’s surface.

Figure 11c presents the CV profiles recorded at 25 mV/s in a 1 M KOH electrolyte with and without different concentrations of methanol (0.5 M, 1 M, 2 M, and 3 M). The CN-CO nanostructure electrode exhibited efficient oxidation, facilitating the transfer of charges during the solid-state reactions involving methanol. The enclosed area of the CV profile amplified with accumulative methanol concentration up to 2 M, designating the excellent MOR performance of the CN-CO nanostructure electrode. This exceptional performance can be attributed to the unique nanostructure features of the CN-CO electrode, particularly the presence of heterostructures between CN and CO. These heterostructures promote ion transport and provide a significantly higher reactive edge site number within the electrode, enhancing the MOR activity. However, beyond a methanol concentration of 2 M, the MOR activity started to decline, suggesting that 2 M is the optimal concentration for the CN-CO nanostructure electrode. The remarkable highest current density of 83.71 mA/cm^2^ was observed at the optimal 2 M methanol concentration, which can be credited to the synergy effects between CN and CO in the nanostructure. The enhanced performance is a result of the combined properties and interactions of these materials. Figure 11d depicts the CV curves at the optimal 2 M methanol concentration recorded at different sweep rates, revealing that the current density rises with increased sweep rates. At 50 mV/s, the achieved current density is 91.93 mA/cm^2^. This indicates the favorable kinetics and rapid transfer of charges at the surface of the CN-CO nanostructure electrode.

**Figure 12:**
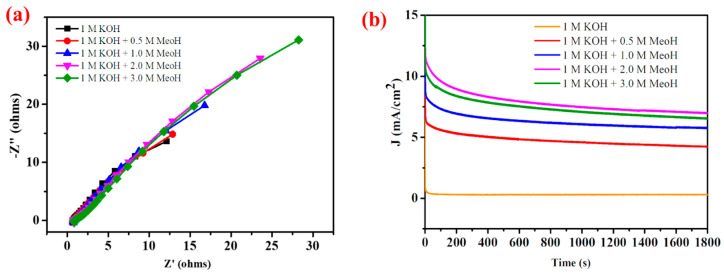
(**a**) Nyquist plots and (**b**) stability of chronoamperometric of the CN-CO nanostructure in Ar-saturated 1 M KOH.

To evaluate the CN-CO nanostructure, electrode stability for MOR was conducted using CA (Figure 12a). CA measurements were performed at 0.45 V Hg/HgO for 30 min, with different methanol concentrations. The results demonstrated the remarkable stability of the CN-CO nanostructure electrode across varying methanol concentrations. The electrode exhibited the highest and stable current density of 8 mA/cm^2^ at the optimal methanol concentration of 2 M. This indicates that 2 M methanol is the most suitable concentration for achieving both stability and optimal performance of the CN-CO nanostructure electrode. Figure 12b presents the EIS response of the electrode. The transfer of charge behavior initially amplified and then diminished as the methanol concentration was raised to 3 M. Minor variations in the electrode resistance were observed, which can be attributed to the unevenness of the electrode surface. However, the EIS spectra exhibited similar shapes under all situations, indicating the superior conductivity and inferior e^−^ transfer resistance of the CN-CO nanostructure. Even when methanol was added above 2 M, the changes observed in the EIS curves were negligible. This highlights the excellent tolerance of the CN-CO nanostructure electrode towards potential electrode poisons. These remarkable stability and tolerance characteristics can be attributed to the heterojunction between CN and CO, as well as the unique 2D/2D features of the nanostructure.

## 4. Conclusions

This study highlights the importance of developing efficient photocatalysts driven by solar energy to combat the global challenge of organic water contamination. The successful synthesis of a novel photocatalyst, CN-CO, was achieved through a simple thermal decomposition process using urea and cobalt nitrate. The resulting CN-CO nanostructures, consisting of CN nanosheets embedded with Co_3_O_4_ material, revealed remarkable photocatalytic activity for the removal of MB pollutants under artificial sunlight. The degradation rate of MB reached an impressive 98.9% after 90 min of exposure, surpassing the rates of pure CN (62.3%) and pure Co_3_O_4_ (89.32%) during the same timeframe. This superior performance can be attributed to the outstanding light absorption capabilities and efficient charge separation features of the CN-CO nanostructures. Additionally, the CN-CO nanostructures exhibited excellent MOR activity, achieving a peak current density of 83.71 mA/cm^2^ at an optimal methanol concentration of 2 M, benefiting from the synergy effects of CN and CO within the nanostructure. Overall, this research offers a straightforward and effective technique for producing CN-based photocatalysts integrated with semiconductor nanosized materials. The outcomes of this study contribute to the advancement of nanostructure design for energy-related uses and offer valuable insights into the progress of efficient photocatalytic materials for addressing environmental challenges.

## Data Availability

Not applicable.

## Supplementary Materials

The following supporting information can be downloaded at: https://www.mdpi.com/article/10.3390/nano13182508/s1, Section S1: Instruments; Section S2: Photocatalytic experiment; Section S3: Electrochemical measurements.
