# Supplementary material for: Enhanced Sunlight-Powered Photocatalysis and Methanol Oxidation Activities of Co3O4-Embedded Polymeric Carbon Nitride Nanostructures
Source: Nanomaterials (Basel). 2023 Sep 6;13(18):2508. doi: 10.3390/nano13182508 (PMC10534687; doi:10.3390/nano13182508)
Supplement: Supplementary file 1 [file nanomaterials-13-02508-s001.zip › nanomaterials-2533184-supplementary.pdf]

S1. Instruments

X-ray diffraction patterns were analyzed on Shimadzu 6100 X-ray diffractometer. The field emission transmission electron microscopy (FETEM) with a FEI Technai G2 F20, and scanning electron microscope (Hitachi SEM-41800) were used for morphology analysis. The x-ray photoelectron spectroscopy (XPS) analysis was performed using a Thermo scientific k-α surface analyzer. The UV-vis diffuse reflectance spectra (DRS) were carried out on a Cary 5000. The infrared spectra were carried out in the spectral range of 400-4000 cm^{-1} using Avator 370 Fourier transform spectrometer. Specific surface area and pore size distribution measured by ASAP 2420 surface area analyzer with degassed for 1h at 150°C. High resolution transimisson electron microscope (HRTEM- FEI company (Titan G2 Chemi STEM Cs probe with EDS windowless (Super-X) model and Physisorption Analyzer (BET-Micromeritics, 3FLEX) were used for morphology and surface area studies.

S2. Photocatalytic experiment

The photoactivity of the all samples were examined by simulating the photocatalytic decolorization of MB under simulated solar light. Specifically, 50 mg of each sample was mixed in 100 mL of MB aqueous solution. The solution was stimulated in the dark for 30 minutes before irradiation. The solution was then irradiated with 300 W MAX-303 lamp with a light intensity of 100 mW/cm^2 as artificial solar light irradiation. Aspirate 5 mL of the solution at given time interims and centrifuge to remove the sample. Spectral measurements were performed on the supernatant after centrifugation.

S3. Electrochemical measurements

Photocurrent and electrochemical impedance spectroscopy (EIS) were conducted on a Biologic SP-200 electrochemical workstation by using a standard three-electrode system. The working electrode was immersed in an aqueous solution of sodium sulfate (0.5 mol L^{-1}), and photocurrent test was performed using 300 W MAX-303 lamp with help of Biologic SP-200 electrochemical workstation. For making of MOR samples: the catalyst ink was prepared by dissolving the active material in ethanol/Nafion (10:1). The ink was then deposited on nickel foam and dried overnight at 80 °C for use as the working electrode. A three-electrode electrochemical cell setup comprising the active material-coated electrodes and a bare nickel foam electrode as the working electrodes, Ag/AgCl and platinum mesh as a reference and counter electrode in 1 M KOH for electrochemical and MOR activity was used.
